# Weighted Geometric Dilution of Precision Calculations with Matrix Multiplication

**DOI:** 10.3390/s150100803

**Published:** 2015-01-05

**Authors:** Chien-Sheng Chen

**Affiliations:** Department of Information Management, Tainan University of Technology, Tainan, 710 Taiwan; E-Mail: t00243@mail.tut.edu.tw; Tel.: +886-6253-2106 (ext. 261)

**Keywords:** weighted geometric dilution of precision (WGDOP), geometric dilution of precision (GDOP)

## Abstract

To enhance the performance of location estimation in wireless positioning systems, the geometric dilution of precision (GDOP) is widely used as a criterion for selecting measurement units. Since GDOP represents the geometric effect on the relationship between measurement error and positioning determination error, the smallest GDOP of the measurement unit subset is usually chosen for positioning. The conventional GDOP calculation using matrix inversion method requires many operations. Because more and more measurement units can be chosen nowadays, an efficient calculation should be designed to decrease the complexity. Since the performance of each measurement unit is different, the weighted GDOP (WGDOP), instead of GDOP, is used to select the measurement units to improve the accuracy of location. To calculate WGDOP effectively and efficiently, the closed-form solution for WGDOP calculation is proposed when more than four measurements are available. In this paper, an efficient WGDOP calculation method applying matrix multiplication that is easy for hardware implementation is proposed. In addition, the proposed method can be used when more than exactly four measurements are available. Even when using all-in-view method for positioning, the proposed method still can reduce the computational overhead. The proposed WGDOP methods with less computation are compatible with global positioning system (GPS), wireless sensor networks (WSN) and cellular communication systems.

## 1. Introduction

Mobile positioning, allowing us to obtain the location information, is becoming increasingly important. In general, the position of the mobile device can be determined by a set of base stations (BSs), satellites, or wireless sensors [1,2]. For example, Global Positioning System (GPS) can provide an accurate position to the user from signals received from the satellites [3]. However, GPS users usually require a particular handset device to calculate their positions when they are fully or partially equipped with a GPS receiver. In addition, GPS-embedded handsets are not functional in buildings or shadowed environments, where direct line-of-sight (LOS) propagation is not achievable. Without the aid of GPS, network-based positioning schemes become a solution, which use time or/and angle measurements from the set of BSs to determine the mobile station (MS) location [2,4]. Wireless sensor networks (WSN) are another solution, which is usually applied to indoor measurements, but the locations of the sensor nodes should be known [1].

Initially, geometric dilution of precision (GDOP) is set as a criterion to select better satellites to meet the desired positioning precision in GPS. In general, a smaller GDOP indicates the positioning accuracy is better. Therefore, the subset with the smallest GDOP is selected for the optimal subset and can be used for positioning [5–7]. Nowadays, GDOP can be used to find the best subset of measurement units. For example, if there are *N* (*N* ≥ 4) usable measurement units, an optimal subset of four measurement units can be found by GDOP. Hence, there will be *C* (*N*, 4) possible GDOP values to be calculated. The GDOP calculation using the matrix inversion method is not suitable here because it requires significant computational operations. When more measurements are used, the complexity of the matrix inversion method increases rapidly [8].

When sufficient measurements are available, the determined optimal measurements with the minimum GDOP can improve the positioning accuracy. Satellite selection techniques can be used with a limited number of channels on the GPS receivers and for application in real time navigation. Due to the limited resources associated with mobile devices and the vast number of measurements required, selecting a subset with the most appropriate measurement units rapidly and reasonably is very critical. The conventional method for calculating GDOP is to use matrix inversion, requiring an enormous amount of computation. Recently, there have been extensive publications discussing how to obtain the GDOP value, or the approximate one, without executing matrix inversion. Some of those papers propose various methods to obtain approximate GDOP [9–12].

In the conventional calculation of GDOP, the variances of range errors are assumed to be identical and independent distributed [13]. However, this assumption contradicts the practical environment, because the error variance from each measurement does not have equivalent amplitude [14]. For example, some GPS satellites are relatively old and have less accuracy. If we assume the same error variance, it will cause positioning errors. A satellite signal is related with the user range accuracy value, carrier-to-noise ratio, elevation angle, and the date of ephemeris, it is unreasonable to be assumed that all these signals have the same variance. Thus, the weighted GDOP (WGDOP) is proposed to overcome this problem [15]. WGDOP is performed by measuring the elevation and the signal-to-noise ratio (SNR) of the receiver [16], so that the location is estimated. A WGDOP minimum algorithm for the combined GPS-Galileo navigation receiver was proposed in [17]. In the past two decades, there has been extensive research on calculating WGDOP to yield superior GPS positioning accuracy [18–25]. Matrix inversion is a widely-used method for calculating WGDOP in most literatures. The trade-off between the quality of performances and the complexity of calculations needs to be seriously concerned. Solving WGDOP algorithm in a short time is critical for real-time applications.

Good measurements, as well as WGDOP algorithm, are key factors that determine the characteristics of wireless positioning systems. When the measurements have different error variances or come from integrated positioning systems, the WGDOP minimum criterion can be used to select the appropriate measurement units to reduce the positioning error. This can present challenges to real time practical applications. The WGDOP calculation using the matrix inversion method is a time and power consuming procedure, and fast calculation of WGDOP is most concerned. WGDOP decreases as the number of satellites increases. Thus, the all-in-view method is adopted by reference [16] and the optimal WGDOP value was obtained through computing all-in-view satellites. With the development of electronics technology, receivers can track many satellites simultaneously and use all the visible satellites to position to improve the accuracy. If the processing capability of the receiver is powerful and the number of visible satellites is not large, the all-in-view method is a good choice to improve the positioning accuracy. However, it takes a considerable computation time. There will be 70–90 satellites in the sky at the same time when Glonass and Galileo reach full operation capability [26], and the visible satellite number can reach 30 in any moment. It thus will become very difficult for people to use the all-in-view procedure for location estimation in the future.

Nowadays, due to the vibrant developments of GPS embedded systems, most smart phones and other wearable electronic devices are already equipped with GPS. Despite the performance increases, these devices still possess limited battery capacities and fast calculation capability. Since the battery life and the processing delay are the main limitations on the performance of mobile phones, GPS service should be provided under the condition of high processing ability and low power dissipation. Hence, for the location-estimating applications, how to derive WGDOP efficiently from a large of measurements sent from different satellites is a critical challenge. Due to the limited resources associated with many mobile devices and the very large number of visible satellites, selecting the desired data from a limited number of suitable satellites is a possible solution [26]. Interpreting the original and complicated WGDOP algorithm as a set of simple approximations greatly reduces the computational complexity. By restricting the scale of measurements, the mobile phones can not only reduce the processor load, but also effectively increase the charged period of batteries. Furthermore, the saved memory space can be used to run other programs.

This paper considers all positioning systems, that is WGDOP can select any suitable measurement units, such as satellites, BSs, or wireless sensors. A smaller WGDOP value is obtained with more usable measurement units. In [15–25], the authors focus on improving the GPS positioning accuracy by WGDOP concepts. Selecting four from 30 satellites would give 27,405 possible subsets. The conventional method for calculating WGDOP is to use the matrix inversion method, which requires a large amount of computation. In order to avoid complicated matrix inversion operations, we presented a novel architecture based on resilient back-propagation (Rprop) to obtain the approximate WGDOP for location estimation in [27]. However, machine learning techniques require computational resources to train a model, which needs troublesome processes and consumes more time. Furthermore, machine learning techniques offer only approximations to the target values. This method suffers from the approximation error and inaccuracy incurred by approximating the inverse matrix in calculating WGDOP. The major drawbacks of neural networks are the need of a training phase with several input-output patterns.

This paper proposes a new hardware design architecture for WGDOP calculations without calculating the inverse matrix. When more than four measurements are available, the proposed formula provides the best computational efficiency. It is a method of multiplication matrices of fixed dimensions. There are many ways to simplify the hardware's complexity for use. The WGDOP scheme we propose does not need of a training phase and the calculation of matrix inversion. It is designed to only need simple matrix multiplication. As a result, this WGDOP calculation with matrix multiplication is easy to implement in practical hardware equipment. In addition, we also provide a computational architecture applied to the computation of more than four measurement units to achieve more advantages in practical application. To further reduce the computational overhead and save battery, satellite selection methods should be used in the GPS receivers in the future, and even in the all-in-view method, the proposed method will contribute to reduce the computation load. The remainder of this paper is organized as follows: Section 2 describes the calculation of GDOP and WGDOP. Section 3 reviews the previous literature for the calculation of WGDOP by the Rprop approximation and closed-form solution methods. The matrix multiplication for WGDOP calculations in the case of *N* (*N* ≥ 4) measurements with different variances are proposed in Section 4. Simulation results are given in Section 5. Lastly, conclusions are given in Section 6.

## 2. Calculation of GDOP and WGDOP

GDOP is commonly used to choose the appropriate subset of measurement units from all available ones by determining the geometric effect of their configurations. It assumes that each measurement has the same error variance. If the subset of the measurement unit has the smallest GDOP value, the subset will be used as the most accurate one for positioning. Define (*x*, *y*, *z*) and (*X_i_*, *Y_i_*, *Z_i_*) are the locations of the user and satellite *s_i_*, respectively. The pseudo distances between satellite *i* and the user could be expressed as *r_i_*. We set *r̂_i_* as *r_i_* at the approximate user position (*x̂*, *ŷ*, *ẑ*) The GDOP is a measure of accuracy for positioning systems and relates closely with the geometry matrix *H*.

(1) $$\text{GDOP}=\sqrt{tr{\left(H^{T}H\right)}^{-1}}$$

where $H=\left[\begin{array}{cccc} e_{11} & e_{12} & e_{13} & 1\\ e_{21} & e_{22} & e_{23} & 1\\ \vdots & \vdots & \vdots & \vdots \\ e_{n1} & e_{n2} & e_{n3} & 1 \end{array}\right]$, $e_{i1}=\frac{\hat{x}-X_{i}}{{\hat{r}}_{i}}$, $e_{i2}=\frac{\hat{y}-Y_{i}}{{\hat{r}}_{i}}$, $e_{i3}=\frac{\hat{z}-Z_{i}}{{\hat{r}}_{i}}$, and ${\hat{r}}_{i}=\sqrt{{\left(\hat{x}-X_{i}\right)}^{2}+{\left(\hat{y}-Y_{i}\right)}^{2}+{\left(\hat{z}-Z_{i}\right)}^{2}}$.

In a real environment, each measurement usually has various variances. The combination of different positioning systems is a significant example. *W* is a diagonal matrix and defined as a weighted matrix:

(2) $$W=\left[\begin{array}{ccccc} 1/\sigma_{1}^{2} & 0 & 0 & 0 & 0\\ 0 & 1/\sigma_{2}^{2} & 0 & 0 & 0\\ 0 & 0 & 1/\sigma_{3}^{2} & 0 & 0\\ 0 & 0 & 0 & \ddots & 0\\ 0 & 0 & 0 & 0 & 1/\sigma_{n}^{2} \end{array}\right]=\left[\begin{array}{ccccc} k_{1} & 0 & 0 & 0 & 0\\ 0 & k_{2} & 0 & 0 & 0\\ 0 & 0 & k_{3} & 0 & 0\\ 0 & 0 & 0 & \ddots & 0\\ 0 & 0 & 0 & 0 & k_{n} \end{array}\right]$$

Where $\sigma_{i}^{2}=1/k_{i}$, *i* = 1,…*n* are variances of measurement errors. According to the formula above, WGDOP is given by the trace of the inverse of the *H^T^WH* matrix:

(3) $$\text{WGDOP}=\sqrt{tr{\left(H^{T}WH\right)}^{-1}}$$

with the minimum WGDOP, the subset is the optimal result we want. In this paper, we apply WGDOP, instead of GDOP for measurement units selection to improve the location accuracy. One of the tasks is to calculate the matrix inversion for all subsets in the conventional method for calculating WGDOP. When the number of dimensions increases, the computation time will increase rapidly. In this paper, we employ a novel scheme to only calculate the matrix multiplication rather than matrix inversion. It will simplify the requirements of hardware computing and be beneficial to the hardware design.

## 3. Related Work

In this section, we discuss different GDOP and WGDOP techniques. The positioning accuracy depends on the suitability of the selection of optimal measurement units. Several methods based on GDOP and WGDOP have been proposed to improve the GPS positioning accuracy [15–25]. These methods need matrix inversion to calculate GDOP and WGDOP. However, the conventional matrix inversion method is rather time consuming and presents a significant computational burden. It increases the computational complexity and is not applicable to real hardware requirements. The handheld GPS devices and mobile phone with GPS chip possess only limited processing ability and power dissipation capability. In order to reduce the computational overhead and improve location performance, many researchers utilize the approximate method or simplify the matrix to avoid the matrix inversion calculations.

### 3.1. Neural Network for GDOP and WDGOP Approximation

#### 3.1.1. Neural Network for GDOP Approximation

It is known from some related literatures that the calculation complexity of an inverse matrix is higher than that of neural networks. A back-propagation neural network (BPNN) is capable of learning and realizing for both linear and nonlinear functions [28]. Simon and El-Sherief used BPNN to obtain a GDOP approximation [9,10]. It is a function approximation technique that uses a training process to find a relationship between inputs and outputs. The BPNN learning process can be considered as a gradient descent method that minimizes some measures. BPNN here is applied to learn the relationship between the entries of a measurement matrix and the eigenvalues of its inverse. Another solution using BPNN for GDOP approximation depending on three other input-output relationships was proposed in [11]. Considering both effectiveness and efficiency, we present two novel architectures based on an alternative artificial neural networks method, namely, the Rprop method, to calculate GDOP [12]. Compared to the traditional BPNN, the Rprop algorithm offers faster convergence and is effective for escaping local minima [29]. The proposed Rprop-based architectures for GDOP always have a high degree of accuracy compared with other architectures. Simulation results show the proposed architectures using Rprop and the matrix inversion method provide nearly identical GDOP value. This can reduce the computational complexity required to compute the matrix inversion for calculating GDOP. The main disadvantage of neural network-based methods is that they can incur approximation errors and need input-output patterns for training.

#### 3.1.2. Neural Network for WDGOP Approximation

Similarly, neural network can also be applied to obtain WGDOP values. The author of this paper are devoted to using the novel Rprop-based architectures to simplify WGDOP calculation [27]. The original four types of GDOP mapping of traditional BPNN is extended to WGDOP based on Rprop and two new mapping architectures are proposed. Instead of calculating the inverse matrix, we collect the elements of related matrix and the desired WGDOP value to train the Rprop neural network in practical applications. Finally, the subset with minimum WGDOP is used to estimate the MS location. From simulation results, the proposed architectures always give a better accuracy, compared with the other architectures for WGDOP approximation. Compared with BPNN, the proposed Rprop-based architecture for Type 6 provides faster convergence on a solution and reduce the number of training iterations [27]. However, the drawback in the neural network-based WGDOP algorithms is the need of a long duration training period with several input-output patterns.

### 3.2. Closed-Form Formulas for GDOP and WDGOP Calculation

#### 3.2.1. Closed-Form Formulas for GDOP Calculation

To achieve both high accuracy and low computational requirements, an efficient closed-form scheme for GDOP has been proposed in [7]. Because the closed-form method obtains the solution directly instead of utilizing an approximation method, the closed-form formulas for GDOP calculations are the most accurate among all. The GDOP formulas can reduce the computational complexity required for computing the matrix inversion. If exactly four measurements are available, the closed-form formula method can provide the best computational efficiency. The disadvantage is that it can be applied for four measurements only.

#### 3.2.2. Closed-Form Formula for WGDOP Calculation

To improve the WGDOP accuracy effectively and efficiently, the author of this paper proposed the closed-form solutions for two WGDOP formations for the case of processing four measurements with different variances and one of the measurements with better accuracy than the others [30]. When exactly four measurements are used, the formula provides the best computational efficiency. The computation load of the closed-form formula is greatly less than that of the matrix inversion method. The proposed efficient formula can provide the exact solution of WGDOP calculation. The WGDOP calculations formula can eliminate the poor geometry influence and reduce the computational overhead.

## 4. GDOP and WGDOP Calculation with Matrix Multiplication

### 4.1. GDOP Calculation with Matrix Multiplication

GDOP is a measurement which is used to select the better satellite set in a GPS system. GDOP is used to determine which measurements are suitable for carrying out the positioning process. In order to reduce the complexity and without having to invert a matrix, reference [31] proposed a simple formula for computing GDOP values. They use Newton's identities to calculate GDOP from the input parameters, including the traces, second and third powers, and the determinant of the measurement matrix.

### 4.2. Proposed WGDOP Calculation with Matrix Multiplication

In practice, the GDOP minimum criteria is not suitable to represent positioning accuracy when the measurements contain different error variances The WGDOP criterion can select more appropriate measurement units by combining GDOP with the weighted scheme. WGDOP has also been proven to have better performance in positioning schemes. The matrix inversion computation is a severe task for solving WGDOP. The training phase has to spend lots of costs, time and hardware and software resources to collect the training data. In some cases, the system is unable to collect sufficient historical information for training. The lack of sufficient training data could often affect the accuracy of prediction. It is very important that how to achieve higher accuracy when the training data is not enough. Therefore, faster convergence and fewer number of training are very important in the neural network field.

Here, we propose an efficient solution of WGDOP calculation with less computation when more than four measurements are available for locating purposes. In order to obtain WGDOP efficiently, the proposed WGDOP scheme with matrix multiplication does not need a training phase. We apply Newton's identities to reduce the calculation complexity and obtain WGDOP value directly. The proposed novel computation architecture for WGDOP is beneficial for hardware design in more than four measurements. In the viewpoint of hardware, many computational rules would be a heavy burden to hardware equipment. Here, we derived the WGDOP value only from matrix multiplication. The proposed WGDOP calculation with matrix multiplication can provide very precise solution of WGDOP approximation and does not incur any approximation errors.

By combing GDOP with weighted scheme, WGDOP can be expressed as Equation (3). In order to guarantee the matrix *H^T^WH* is a symmetric one in later operation, we refreshed the formulation above:

(4) $$\text{WGDOP}=\sqrt{tr{\left(H^{T}WH\right)}^{-1}}=\sqrt{tr{\left(H^{T}W^{\frac{1}{2}}W^{\frac{1}{2}}H\right)}^{-1}}=\sqrt{tr{\left(W^{\frac{1}{2}}HH^{T}W^{\frac{1}{2}}\right)}^{-1}}$$

#### 4.2.1. Four Measurement Units

We have the measurement matrix *M* given that:

(5) $$\begin{array}{l} M=W^{\frac{1}{2}}HH^{T}W^{\frac{1}{2}}=\left[\begin{array}{cccc} \sqrt{k_{1}} & 0 & 0 & 0\\ 0 & \sqrt{k_{2}} & 0 & 0\\ 0 & 0 & \sqrt{k_{3}} & 0\\ 0 & 0 & 0 & \sqrt{k_{4}} \end{array}\right]\;\left[\begin{array}{cccc} e_{11} & e_{12} & e_{13} & 1\\ e_{21} & e_{22} & e_{23} & 1\\ e_{31} & e_{32} & e_{33} & 1\\ e_{41} & e_{42} & e_{43} & 1 \end{array}\right]\\ \left[\begin{array}{cccc} e_{11} & e_{21} & e_{31} & e_{41}\\ e_{12} & e_{22} & e_{32} & e_{42}\\ e_{13} & e_{23} & e_{33} & e_{43}\\ 1 & 1 & 1 & 1 \end{array}\right]\;\left[\begin{array}{cccc} \sqrt{k_{1}} & 0 & 0 & 0\\ 0 & \sqrt{k_{2}} & 0 & 0\\ 0 & 0 & \sqrt{k_{3}} & 0\\ 0 & 0 & 0 & \sqrt{k_{4}} \end{array}\right] \end{array}$$

and following the relationship:

(6) $$e_{i1}^{2}+e_{i2}^{2}+e_{i3}^{2}=1,i=1,2,3,4$$

We have a simple form of trace and a guaranteed symmetric matrix *M*:

(7) $$M=W^{\frac{1}{2}}HH^{T}W^{\frac{1}{2}}=\left[\begin{array}{cccc} 2k_{1} & \sqrt{k_{1}k_{2}}B_{12} & \sqrt{k_{1}k_{3}}B_{13} & \sqrt{k_{1}k_{4}}B_{14}\\ \sqrt{k_{1}k_{2}}B_{12} & 2k_{2} & \sqrt{k_{2}k_{3}}B_{23} & \sqrt{k_{2}k_{4}}B_{24}\\ \sqrt{k_{1}k_{3}}B_{13} & \sqrt{k_{2}k_{3}}B_{23} & 2k_{3} & \sqrt{k_{3}k_{4}}B_{34}\\ \sqrt{k_{1}k_{4}}B_{14} & \sqrt{k_{2}k_{4}}B_{24} & \sqrt{k_{3}k_{4}}B_{34} & 2k_{4} \end{array}\right]$$

where *B_ij_* = *e_i_*_1_*e_j_*_1_ + *e_i_*_2_*e_j_*_2_ + *e_i_*_3_*e_j_*_3_ +1, 1 ≤ *i* < *j* ≤ 4.

Utilizing the leaner algebra method, WGDOP can be written as:

(8) $$\text{WGDOP}=\sqrt{tr{\left(H^{T}WH\right)}^{-1}}=\sqrt{{\lambda_{1}}^{-1}+{\lambda_{2}}^{-1}+{\lambda_{3}}^{-1}+{\lambda_{4}}^{-1}}$$

Defining the following functions:

(9a) $$p_{1}(\lambda )=\lambda_{1}+\lambda_{2}+\lambda_{3}+\lambda_{4}=\text{trace}(H^{T}WH)=\text{trace}(M)$$

(9b) $$p_{2}(\lambda )=\lambda_{1}^{2}+\lambda_{2}^{2}+\lambda_{3}^{2}+\lambda_{4}^{2}=\text{trace}[{\left(H^{T}WH\right)}^{2}]=\text{trace}[M^{2}]$$

(9c) $$p_{3}(\lambda )=\lambda_{1}^{3}+\lambda_{2}^{3}+\lambda_{3}^{3}+\lambda_{4}^{3}=\text{trace}[{\left(H^{T}WH\right)}^{3}]=\text{trace}[M^{3}]$$

(9d) $$p_{4}(\lambda )=\lambda_{1}\cdot \lambda_{2}\cdot \lambda_{3}\cdot \lambda_{4}=\det(H^{T}WH)$$

After verify that the measurement matrix is symmetric, we can use Newton's identity for reduction Equation (8). The WGDOP formula is thus obtained:

(10) $$\text{WGDOP}=\sqrt{\frac{1}{3p_{4}}(\frac{1}{2}{p_{1}}^{3}-\frac{3}{2}p_{1}p_{2}+p_{3})}$$

**Figure 1.**
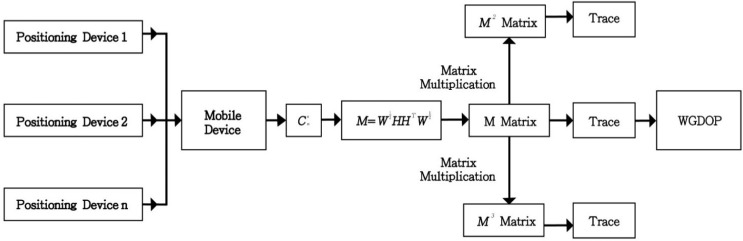
Block diagram of WGDOP process.

Based on the operation rules above, we derive the WGDOP value only from calculating Equation (10). The formula values of *p_1_*, *p_2_*, *p_3_* requires matrix multiplication and *p_4_* requires only simple arithmetic. Because the measurement matrix *M* is a symmetric matrix, the components of *M* present a symmetric form. We can determine that the matrix of *M^2^*, *M^3^* are symmetric matrices also, so only the upper half components of matrix *M*, *M^2^*, *M^3^* needs to be calculated. Then the WGDOP can be solved by the proposed matrix multiplication scheme for four measurement units. According to the above described matrix multiplications, we can easily find the value of the WGDOP and avoid the calculation of the matrix inverse. We summarize WGDOP formula process and present it in Figure 1.

The steps for positioning in GPS, WSN, and cellular communication systems are listed as follows:

(1) After a mobile device receives the signals from the positioning device, we arbitrarily select *m* measurements among *n* measurements to generate different subsets, thus the *n* measurements are classified into *C* (*n*, *m*) possible subsets.
(2) The optimal subset is often obtained by minimizing the WGDOP value.
(3) The subset with the smallest WGDOP value is selected as the optimal measurement constellation subset:
  (a) First, we calculate the measurement matrix *M*.
  (b) Then the matrix of *M*, *M^2^*, and *M^3^* are obtained by matrix multiplication method which is acceptable to most hardware. The determinants and trace values are calculated.
  (c) Finally, these parameter values can be used to calculate WGDOP value. The values of variance 1/*k_i_*, *i* = 1, 2, 3, 4, can be treated as constants, which are previous assumed for the calculation of Equation (2).
(4) Finally, select the optimal subset of *m* measurements to estimate the location solution. From Table 1, the matrix multiplication scheme needs only 134 additions, 212 multiplications, one division and one square root.

#### 4.2.2. Five or More Measurement Units

Many scholars have proposed WGDOP calculation methods only using in the case of exactly four measurement units. Most of the existing literatures and patents only discuss the derivations of the formula in the situation of selecting four measuring devices. When the number of measurements increases, WGDOP is decreasing. More than four measurements are selected because more measurements can obtain a higher positioning accuracy. Here, we expand the WGDOP computational formulas, so that it can be successfully applied in the case of more than four measurements. This calculation device greatly enhances WGDOP computing applications in practice. If the number of measurement units is *N*, the dimension of measurement matrix *M* is *N* × *N*. When more than four measurements are available for location purposes, Equations (9) and (10) can still be adopted to describe the calculation forms of *p*_1_(*λ*), *p*_2_(*λ*), *p*_3_(*λ*), *p*_4_(*λ*)and WGDOP.

In this method, the rest formula for *N* (*N* ≥ 5) may be deduced by analogy. Reduction by Newton's identities simplifies WGDOP formula and satisfies the needs of obtaining various parameters in the formula. Finally, we can obtain the WGDOP value for positioning. From Table 2, the matrix multiplication scheme with five measurements needs only 221 additions, 326 multiplications, one division, and one square root. Simultaneously, the computational complexity of the proposed WGDOP criteria can be reduced by a matrix multiplication scheme for measurement units *N* (*N* ≥ 5). These results show that the WGDOP methods proposed in this paper have low computational complexity and are easy to implement in the hardware equipment because of the simplicity of its operation rules. The proposed WGDOP formula with matrix multiplication does not limit the number of measurement units selected.

## 5. Simulation Results

We consider a center hexagonal cell with six adjacent hexagonal cells of the same size. Each cell has a radius of 5000 m and the MS locations are uniformly distributed in the center cell [32]. There are seven BSs in cellular communication systems. The serving BS, that is, BS_1_, is located at (0, 0, 0.2 km). The heights of the other six BSs, BS_2_, BS_3_, BS_4_, BS_5_, BS_6_, and BS_7_, are 0.15 km, 0.14 km, 0.16 km, 0.12 km, 0.11 km, and 0.13 km, respectively. The height of MS is assumed to be at uniformly distributed over (0, 0.06 km). The NLOS propagation model is based on the uniformly distributed noise model [33], in which the TOA measurement error is assumed to be uniformly distributed over (0, *U_i_*), for *i* = 1, 2, …7 where *U_i_* is the upper bound. The variables are chosen as follows: *U*_1_ 0.2 km, *U*_2_ = 0.4 km, *U*_3_ = 0.35 km, *U*_4_ = 0.7 km, *U*_5_ = 0.3 km, *U*_6_ = 0.5 km and *U*_7_ = 0.35 km. The reciprocal of the square root of an upper bound of the NLOS errors is set to be diagonal elements of the weight matrix *W*. Therefore, the previously proposed BSs selection criterion chooses the serving BS first and combines it with three optimal measurements to form a subset [27]. The number of the subsets is reduced from 35 ( $C_{4}^{7}$) to 20 ( $C_{3}^{6}$) and the calculation load can be relaxed. The subset of the serving BS and three optimal BSs with minimum WGDOP is used to estimate the mobile station (MS) location. The WGDOP residual is defined as the difference between the WGDOP value by matrix inversion and the estimation methods.

**Figure 2.**
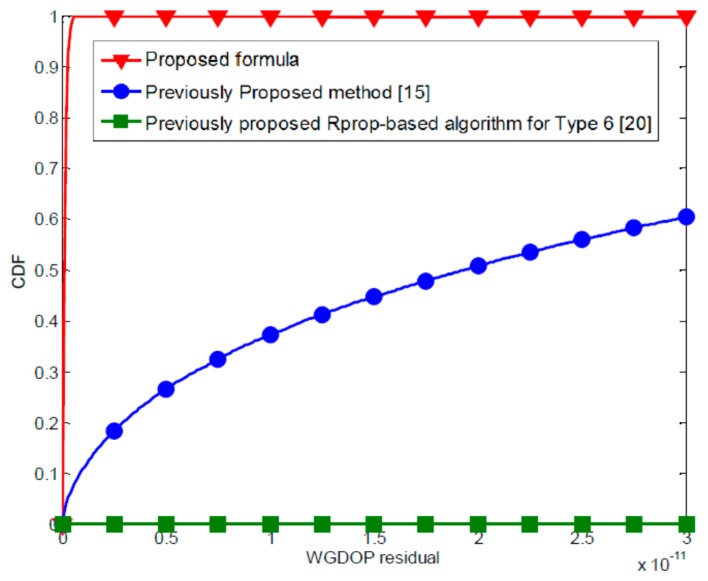
The CDFs curves of WGDOP residual for all methods when four measurements are available.

In order to verify the superior properties of the proposed formula, we compare the results of WGDOP calculation accuracy for all methods and matrix inversion method. Figure 2 shows cumulative distribution functions (CDFs) of the average WGDOP residual for the proposed formula, the previously proposed method [30] and Rprop-based algorithm for Type 6 [27] when four measurements are available. From simulation results, the proposed method and matrix inversion method provide nearly identical WGDOP estimation. The average WGDOP residual of the proposed formula is 2.024245 × 10^−12^. The proposed efficient formula provides very precise solution of WGDOP calculation. Table 3 shows average WGDOP residual for all methods. Compared to other previously proposed methods, the proposed method always yields much better WGDOP residual than the previously proposed methods.

**Figure 3.**
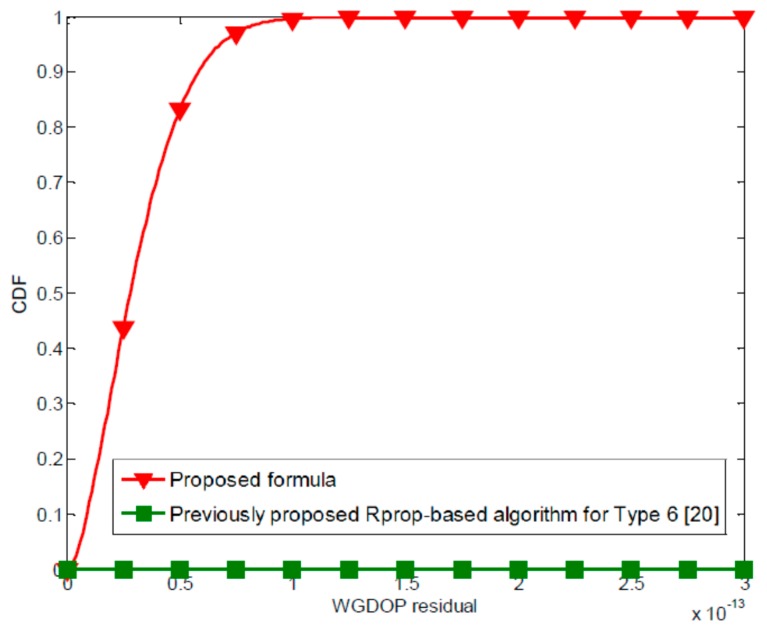
The CDFs curves of WGDOP residual for all methods when five measurements are available for location purpose.

The improvement in WGDOP accuracy using the proposed formula can also be seen, the CDF curves of the average WGDOP residual for all methods when five BSs are available for location purposes, as shown in Figure 3. The proposed formula can give much better WGDOP estimation as compared with the other previously proposed methods. Table 4 shows the average WGDOP residual for all methods under the constraint that the MS can be heard by five BSs. The average WGDOP residual of the proposed formula is 3.110480 × 10^−3^. It was observed that the proposed formula gives the best performance among the previously proposed methods.

## 6. Conclusions

Traditionally, the concept of GDOP is commonly used to determine the geometric effect of GPS satellite configurations. It is desirable to select a set of satellites with GDOP as small as possible. When the measurements have different error variances or in integrated positioning systems, WGDOP is appropriate to select the measurement units to reduce the positioning error. The matrix inversion method guarantees the optimal subsets but with a significant computational burden. To further reduce the complexity, a novel matrix multiplication scheme is proposed to compute WGDOP. The matrix multiplication scheme not only avoids complex computations, but is also easy to design in hardware architecture. The proposed WGDOP calculation with matrix multiplication does not need to implement a training phase and the calculation of matrix inversion. It is designed to only need simple matrix multiplication in which the *a priori* error information of each measurement is not the same. In addition, we also provide a calculation device applied on the computation of more than four positioning devices. No matter how many measurements are used, the proposed WGDOP scheme has the best computational efficiency. The proposed WGDOP calculation with matrix multiplication provides very precise WGDOP calculation solutions and is very suitable to implement in practical hardware equipment.

## Figures and Tables

**Table 1.** The complexity of WGDOP calculation when the four measurements with different error variances.

	**Additions**	**Multiplications**	**Division**	**Square Root**
*M*	18	34	0	0
*M*^2^	30	54	0	0
*M*^3^	30	50	0	0
det(*M*)	45	68	0	0
WGDOP	11	6	1	1

Total	134	212	1	1

**Table 2.** The complexity of WGDOP calculation when the five measurements with different error variances.

	**Additions**	**Multiplications**	**Division**	**Square Root**
*M*	30	55	0	0
*M*^2^	60	98	0	0
*M*^3^	60	90	0	0
Det(*M*)	60	77	0	0
WGDOP	11	6	1	1

Total	221	326	1	1

**Table 3.** Comparison of average WGDOP residual for all methods when four measurements are available.

**Method**	**Average WGDOP Residual**
Proposed formula	2.024245 × 10^−12^
Previously proposed method [30]	1.694013 × 10^−11^
Previously proposed Rprop-based algorithm for Type 6 [27]	0.238554

**Table 4.** Performance comparison of average WGDOP residual for all methods when five measurements are available for location purpose.

**Method**	**Average WGDOP Residual**
Proposed formula	3.110480 × 10^−14^
Previously proposed method [30]	Cannot calculate
Previously proposed Rprop-based algorithm for Type 6 [27]	0.16803456595604
